# Chlorogenic Acid: A Promising Strategy for Milk Preservation by Inhibiting *Staphylococcus aureus* Growth and Biofilm Formation

**DOI:** 10.3390/foods13244104

**Published:** 2024-12-18

**Authors:** Xiaoyan Yu, Yufang Li, Xue Yang, Jinze He, Wenhuan Tang, Yunmei Chai, Zuyan Duan, Wenjie Li, Dan Zhao, Xuefeng Wang, Aixiang Huang, Hong Li, Yanan Shi

**Affiliations:** 1 College of Food Science &Technology, Yunnan Agricultural University, Kunming 650201, China; yxy15087959659@163.com (X.Y.); yufangfangli@126.com (Y.L.); 18428000763@163.com (X.Y.); hejinze1123@126.com (J.H.); 17806901674@163.com (W.T.); yunmeichai@163.com (Y.C.); 13529522760@163.com (Z.D.); liwenjie2436@126.com (W.L.); minyanzhiliu@163.com (D.Z.); wangxuefeng2870@126.com (X.W.); aixianghuang@126.com (A.H.); 2 Yunnan College of Modern Coffee Industry, Yunnan Agricultural University, Kunming 650201, China

**Keywords:** *S. aureus*, chlorogenic acid, anti-biofilm, preservative, protein interaction, metabolomics, milk

## Abstract

Chlorogenic acid (CGA), a polyhydroxy phenolic acid, has been extensively studied for its antimicrobial properties. *Staphylococcus aureus* (*S. aureus*) threatens food safety by forming biofilms. This study aimed to investigate the mechanism of CGA against *S. aureus* and its biofilm. The anti-bacterial activity of CGA was assessed using crystal violet staining, TEM, SEM, a CLSM, and using metabolomics and molecular docking to elucidate the mechanism. The results indicated that the minimum inhibitory concentration of CGA against *S. aureus* was 2.5 mg/mL. CGA disrupts the integrity of bacterial cell membranes, leading to increased hydrophobicity, morphological changes, scattering, and reduced spreading. This disruption decreases biofilm adhesion and bacterial count. Metabolomics and molecular docking analyses revealed that CGA down-regulates key amino acids. It forms hydrogen bonds with penicillin-binding protein 4 (PBP4), Amidase, glutamate synthetase B, and glutamate synthetase A. By inhibiting amino acid metabolism, CGA prevents biofilm formation. CGA interacts with amino acids such as aspartic acid, glutamine, and glutamate through hydroxyl (-OH) and carbonyl (-C=O) groups. This interaction reduces cell viability and biofilm cohesion. The novel findings of this study, particularly the extension of the shelf life of pasteurized milk by inhibiting *S. aureus* growth, highlight the potential of CGA as a promising anti-biofilm strategy and preservative in the dairy industry.

## 1. Introduction

*Staphylococcus aureus* (*S. aureus*) is a common foodborne pathogen worldwide and exists as planktonic cells and biofilms. The ability of biofilm formation comprises the advantage of *S. aureus* to resist adverse environments and spread virulence [1,2]. Studies have found that viable cells in *S. aureus* biofilms exhibit higher pathogenicity and transmission ability. Living cells in dry biofilms can spread rapidly in the environment and cause new infections. This transmission ability is closely related to the structure and composition of biofilm [3]. *S. aureus* can enter the food chain and contaminate food raw materials such as milk, meat, eggs, and their by-products [3]. In addition to *S. aureus*, other pathogenic bacteria, such as *Salmonella*, *Listeria*, *Escherichia coli*, etc., are also associated with foodborne diseases related to dairy products [4]. Studies have shown that approximately 241,000 people in the United States become ill each year from eating foods contaminated with *S. aureus*, most of which are associated with dairy products [5]. This is a global problem, and the presence of *S. aureus* in dairy products has been reported in many countries [2,6]. Currently, biofilm-forming pathogenic bacteria such as *S. aureus* are considered to be the major cause of foodborne illness, which has become a major challenge for the food industry.

The *S. aureus* DC.RB-015 strain, isolated from 124 *Rubing* cheeses from various regions of Yunnan Province, China, has biofilm-forming ability and has multiple drug resistance genes, making it highly resistant to penicillin, oxacillin, erythromycin, clindamycin, tetracycline, and cefoxitin [3,4]. The biofilm structure provides shelter and nutrients for microorganisms, ensuring their adaptability to nasty growth conditions. In addition, biofilms can inhibit the entry of antibiotics into bacterial cells, such as beta-lactam antibiotics. The multi-layered structure of the biofilm can physically block their penetration and further increase their drug resistance, making the biofilm formed by pathogenic bacteria such as *S. aureus* a serious threat to food safety and public health [2]. The addition of artificial food preservatives is one of the main traditional methods of controlling food microbial contamination. Some studies have shown that chemical preservatives may trigger allergic reactions, especially in sensitive people. Long-term intake may lead to chronic health problems such as endocrine disruption and decreased immune system function [6]. Prolonged exposure to some chemical preservatives can also lead to nerve damage [7].

Currently, there is increasing research interest in finding plant-derived environmentally friendly antibacterial agents [8]. Chlorogenic acid (CGA, C_16_H_18_O_9_) is a naturally occurring phenolic acid and is abundant in green coffee beans, *Eucommia ulmoides Oliv*, and other plants [9,10,11]. It has significant antibacterial and biofilm inhibition properties, which are mediated via various mechanisms. (i) CGA increases the permeability of the outer and plasma membranes by disrupting the cell membrane (CM) of *S. aureus* and *Shigella dysenteriae*, resulting in the loss of the bacterial barrier function and exerting its antibacterial activity [12,13]. (ii) With regard to cell wall (CW) destruction, after the destruction of CM due to the reduction in CM structural support and the decrease in CW hardness, *Escherichia coli* was destroyed due to CW deformation under external pressure [14]. (iii) With regard to genetic destruction, CGA was reported to down-regulate the expression of the *treC* gene to inhibit capsular polysaccharide (CPS) synthesis, which negatively affects the biofilm formation ability of *Klebsiella pneumoniae* [15]. (iv) With regard to inhibition–resistance, CGA down-regulates the resistance-related proteins (Tsr, Tar, CheA, and CheW), outer membrane (OM) porin F (Omp F), and flagellin (FliC) of multidrug-resistant *Escherichia coli*, thereby inducing bacterial flagella shedding and reducing biofilm stability [14,16]. (v) With regard to the disruption of intracellular communication, CGA inhibits the biofilm formation of *Pseudomonas aeruginosa (P. aeruginosa)* by forming hydrogen (H) bonds with three quorum-sensing (QS) receptors LasR, RhlR, and PqsR, thus interfering with the binding of signal molecules to their receptors. This further down-regulates the expression of the QS system and its related genes, and it also reduces the secretion of OM vesicles and extracellular DNA in *P. aeruginosa* [17,18].

Although CGA can effectively inhibit bacterial growth and biofilm formation, there are limited studies on its role and underlying mechanisms in the inhibition of *S. aureus* biofilm formation. In this study, we evaluated the antibacterial effect of CGA on *S. aureus* in the early stage of biofilm formation by measuring the growth curve, hydrophobicity, spreading, and morphological features of *S. aureus*. Additionally, the inhibitory effect of CGA on *S. aureus* biofilm formation was evaluated by biofilm cell quantification, laser confocal microscopy, and fluorescence microscopy. Furthermore, the effect of CGA on the metabolic activity of *S. aureus* was analyzed by metabolomics to determine the mechanism underlying its antibacterial and anti-biofilm effects. In addition, molecular docking and molecular electrostatic potential were combined to further explore the inhibitory effects of CGA against *S. aureus*. Lastly, the bactericidal effect of CGA on *S. aureus* was also evaluated in pasteurized milk. This study expands the possibility of CGA as a natural bacteriostatic agent in the food industry and provides a theoretical basis and candidate target molecules for the development of novel anti-*S. aureus* agents.

## 2. Materials and Methods

### 2.1. Bacterial Strain and Cultural Conditions

The *S. aureus* DC.RB-015 strain was provided by Yunnan Provincial Center for Disease Control and Prevention (Kunming, China) and cultured in Luria–Bertani (LB) (Guangdong Huankai Microbial Technology Co., Ltd., Guangzhou, China) broth at 37 °C to logarithmic growth phase. For subsequent experiments, the bacterial suspension was adjusted to 10^6^ CFU/mL by diluting with 20 mM phosphate-buffered saline (PBS, pH 7.4). CGA (≥98.0%, Cat.No.c8960, CAS:327-97-9) was purchased from Soleibao Technology Co., Ltd. (Beijing, China). CGA was dissolved in sterile deionized water.

### 2.2. Analysis of the Antibacterial Activity of CGA on S. aureus in the Early Stage of Biofilm Growth

#### 2.2.1. Determination of Minimum Inhibitory Concentration (MIC)

The MIC of CGA against *S. aureus* was determined as described by Sun et al. [19] with slight modifications. In total, 50 μL of fresh LB medium, 10 μL of bacterial suspension (10^6^ CFU/mL), and 40 μL CGA solutions were added to a 96-well plate. The final concentrations of CGA in the solution were 2.0, 2.5, 3, 3.5, and 4.0 mg/mL, respectively. An equal volume (40 μL) of sterile water was added to the blank control group. The culture plates were incubated at 37 °C for 18 h, and the optical density at 600 nm (OD_600 nm_) was measured by a microplate reader (Biotek, Winooski, VT, USA).

#### 2.2.2. Growth Curve Analysis

Growth curve analysis was conducted according to the method described by Yang et al. [20]. Briefly, the CGA solution was added to 10^6^ CFU/mL *S. aureus* suspension to the final mass concentration of 2.5 mg/mL and 1.25 mg/mL. An equal volume of sterile water was added to the blank control group. The cells were incubated at 37 °C for 24 h in a constant temperature incubator, and samples were obtained every 2 h to determine the OD_600 nm_ value to generate the growth curve.

#### 2.2.3. Determination of Cell Surface Hydrophobicity of Bacteria

Bacterial cell surface hydrophobicity was determined as described by Hou et al. [21] with slight modifications. The logarithmic phase bacteria were collected by centrifugation, washed thrice with PBS, and resuspended with 0.1 mol/L KNO_3_ solution. Thereafter, an equal volume of CGA solutions of varying concentrations was added to the bacterial suspension to the final concentration of 0.625 mg/mL, 1.25 mg/mL, and 2.5 mg/mL. An equal volume of sterile water was added to the control group. The samples were incubated at 37 °C for 12 h, and the OD_405_ value (A0) was measured. Thereafter, 1.2 mL of bacterial suspension was aspirated, mixed with 200 μL of hexadecane, vortexed for 2 min, and incubated for 15 min until two-phase separation was obtained. The aqueous phase was then carefully aspirated, and the OD_405_ value (A1) was measured. Hydrophobicity is calculated as follows.

$$Hydrophobicity(\%)=(1-\frac{A1}{A0})\times 100$$

#### 2.2.4. Determination of CM Integrity

The effect of CGA on *S. aureus* cells was observed by scanning electron microscopy (SEM), as described in a previous study by Ren et al. [22], with minor modifications. *S. aureus* in LB broth was treated with 2.5 mg/mL of CGA at 37 °C for 14 h, and the control group was added with equal volume of sterile water. The samples were centrifuged and washed three times with PBS. The cells were then fixed with 2.5% glutaraldehyde solution at 4 °C for 12 h and centrifuged at 10,000× *g* for 2 min at room temperature to remove the fixative. Subsequently, the cells were washed thrice with PBS and dehydrated using an ethanol gradient (30%, 50%, 70%, 80%, 90%, and 100% for 10 min each). The sample was treated twice with 100% ethanol and subsequently resuspended with 100% ethanol. A certain amount of heavy suspension was dried on the SEM stub and sprayed with gold for SEM (Hitachi, Tokyo, Japan) observation.

The effect of CGA on *S. aureus* cells was observed by transmission electron microscopy (TEM), as described previously [23]. The bacterial samples were pretreated as described above for SEM observation. The bacteria were fixed with 2.5% glutaraldehyde solution for 12 h, fixed with 1% osmic acid (pH 7.2) solution for 2 h, washed with PBS 3 times, and centrifuged to obtain bacterial precipitation. 

The samples were then dehydrated with 30%, 50%, 70%, 80%, 90%, and 100% ethanol for 10 min each. Thereafter, the cells were soaked in an embedding agent (Epoxy resin 618) at room temperature for 2 h and incubated at 60 °C for 48 h. The slices were immersed in uranium acetate solution (pH 4.5) for 3 h at room temperature. Subsequently, the sections were transferred to a lead citrate solution for secondary staining. After staining, the samples were washed with deionized water and dried in air or low-temperature oven. The sections were observed by TEM (JEOL, Tokyo, Japan).

#### 2.2.5. Cell Viability Assay

Cells were stained using a live/dead cell staining kit (Invitrogen, California, USA). *S. aureus* was cultured to the logarithmic phase and harvested by centrifugation (4000× *g* at 4 °C for 15 min) and then resuspended in fresh LB broth (10^6^ CFU/mL). CGA was added to the bacterial suspension at a final concentration of 2.5 mg/mL. The control group was added with sterile deionized water and incubated at 37 °C for 12 h. After 12 h, the cells (10,000× *g*) were harvested by centrifugation 4 °C; 15 min), washed three times with 0.1 M PBS buffer (pH 7.2), and resuspended in PBS buffer. Then, PI with a concentration of 60 μM and 10 μM SYTO 9 were mixed in equal volume, and an appropriate amount of dye mixture was added to the bacterial suspension to ensure that the dye was fully mixed with the bacteria. The mixture of bacteria and dyes was incubated in the dark at room temperature for 15 min. After the cells were washed with PBS and resuspended, 10 uL was dropped into the slide and covered with a coverslip. The cell staining was observed by laser scanning confocal microscope (CLSM, Leica Microsystems, Wetzlar, Germany) and the images were collected.

#### 2.2.6. Determination of Bacterial Spreading

The sliding spreading assay was performed according to the method reported by Bai et al. [24]. LB plates containing 0, 1.25 mg/mL, 2.5 mg/mL, and 5 mg/mL of CGA and 1.2% agar were prepared. Upon solidifying, the water on the medium surface was dried, and 5 μL of *S. aureus* (10^6^ CFU/mL) was added to the center of the plate. The plates were placed in an incubator at 37 °C for 24 h and bacterial growth was observed.

### 2.3. Analysis of the Biofilm-Inhibiting Activity of CGA on S. aureus

#### 2.3.1. Determination of *S. aureus* Biofilm Formation

The effect of CGA on the biofilm formation of *S. aureus* was observed by crystal violet staining [8]. For this, in 96-well plates, 20 μL of *S. aureus* suspension (10^6^ CFU/mL), 80 μL of sterile LB liquid medium, and 100 μL of CGA solution were added to make the final concentration of CGA solution 0.625 mg/mL, 1.25 mg/mL, 2.5 mg/mL, and 5 mg/mL. The control group used sterile deionized water instead of CGA solution. The 96-well plates were incubated in a 37 °C incubator for 48 h. Thereafter, the wells were washed thrice with sterile water to remove the planktonic cells, and the residual biofilms were air-dried. Subsequently, 150 μL of methanol was added to the well to immobilize the biofilm. After 15 min, the methanol was removed and the biofilm was air-dried. After staining with 150 μL 0.1% crystal violet solution for 15 min, the biofilm was washed with sterile water and waited for drying. Then, the biofilm was dissolved in 150 μL 95% ethanol. The absorbance at 590 nm was measured using a microplate reader.

#### 2.3.2. Determination of *S. aureus* Biofilm Structure

The structure of *S. aureus* biofilm was observed using an inverted fluorescence microscope, as described previously [25], with slight modifications. In the 48-well plate containing sterile cell-attached slides (φ 9 mm), 100 μL of *S. aureus* bacterial suspension (10^6^ CFU/mL), 400 μL of sterile LB liquid medium, and 500 μL of CGA solution were added to give a final concentration of CGA solution of 2.5 mg/mL. In the control group, the CGA solution was replaced with sterile deionized water. The 48-well plate was incubated at 37 °C for 48 h to form a biofilm. The cells were washed with PBS to remove the planktonic cells, and the residual biofilms were treated with 10 μg/mL 4′,6-diamidino-2-phenylindole (DAPI) for 20 min in the dark. Lastly, the biofilm structure was observed under an inverted fluorescence microscope (Carl Zeiss AG, Oberkochen, Germany).

The anti-biofilm effect of CGA on *S. aureus* was also determined using a CLSM [26]. Biofilm samples were prepared for CLSM analysis. After removing the planktonic cells in the cell-attached slides (φ 9 mm) with PBS, the cells were stained with 5 μM SYTO 9 (Thermo Fisher Scientific, Waltham, MA, USA) and 30 μM PI (Xiya, Henan, China) at 37 °C in dark for 30 min. The cell-attached slides were placed on the glass slides, and an appropriate amount of anti-fluorescence attenuation sealing agent was added. The sample was covered with a cover slip, gently pressed to exclude bubbles, and solidified for 12 h. The sealed samples were placed on the CLSM stage (Leica Microsystems, Wetzlar, Germany), and the microscope parameters were adjusted for imaging.

### 2.4. Metabolomics Analysis

#### 2.4.1. Extraction of *S. aureus* Metabolites

For this purpose, 40 mL *S. aureus* suspension was treated with 2.5 mg/mL CGA at 37 °C for 12 h, and the control group was treated with sterile deionized water. At 4 °C, cells were collected by centrifugation at 3000 rpm for 10 min. After resuspension with PBS solution, 200 μL bacterial solution (about 10^6^ bacteria) was mixed with 1000 μL of extraction solution (methanol–acetonitrile–aqueous solution, 2:2:1 (*v*/*v*)), the extraction solution contained deuterated internal standards (Shanghai Anpu Experimental Technology Co., Ltd., Songjiang, China), and the mixed solution was vortexed for 30 s. Afterward, 2 homogenization beads were added and homogenized for 4 min (35 Hz) and then transferred to an ice-water bath to sonicate for 5 min. (Repeat 3 times.) The samples were then allowed to thaw at room temperature and vortexed for 30 s. This freeze-thaw cycle was repeated three times. Then, the samples were sonicated for 10 min in a 4 °C water bath and incubated for 1 h at −40 °C to precipitate proteins. The samples were centrifuged at 12,000 rpm (RCF = 13,800× *g*, R = 8.6 cm) for 15 min at 4 °C. The supernatant was transferred to a fresh glass vial for liquid chromatography–tandem mass spectrometry (LC-MS/MS) analysis.

#### 2.4.2. LC–MS/MS Analysis and Data Processing

The target compounds were separated by Waters ACQUITY UPLC BEH Amide (2.1 mm × 50 mm, 1.7 μm) liquid chromatography column and analyzed by Vanquish (Thermo Fisher Scientific, Waltham, MA, USA) ultra-high-performance liquid chromatography combined with Exploris 120 mass spectrometer (Thermo Fisher Scientific, Waltham, MA, USA) for LC-MS/MS analysis. The conditions for LC–MS/MS were based on a previous report [27]. The A phase was composed of 25 mmol/L ammonium acetate and 25 mmol/L ammonia, while the B phase was acetonitrile. The temperature of the automatic injector was set to 4 °C, and the injection volume was 2 μL. Mass spectrometry detection of metabolites was performed by electrospray ionization in positive and negative ion modes to ensure optimal identification. Data processing referred to the method of Zhou et al. [28]. The raw data were transformed into mzXML format by ProteoWizard software 3.0, and the metabolites were identified by R package and Biotree DB (V3.0) for visualization.

### 2.5. Molecular Docking Analysis

The crystal structure of the *S. aureus* Amidase (AM; PDB ID: 4knk) protein used for docking was downloaded from the PDB database. The crystal structures of glutamate synthetase B (gltB; PDB ID: 1EA0), glutamate synthetase A (glsA; PDB ID: 3CZD), and penicillin-binding protein 4 (PBP4; PDB ID: 1TVF) were downloaded from the AlphaFold server (https://alphafoldserver.com). The 3D structure of CGA was downloaded from the PubChem database, and energy minimization was performed under the MMFF94 force field.

The AutoDock Vina 1.2.3 software was used for molecular docking. PyMol V2.5.52 was used for receptor protein processing. ADFRsuite V1.03 was used to convert all the processed small molecules and receptor proteins into the PDBQT format. The docking box was placed around the protein structure. The global search was set to 32, and the remaining parameters were kept at the default settings. The docking conformation with the highest output score was considered to be the binding conformation. Lastly, PyMol 2.5.5 was used to visualize the molecular docking results.

### 2.6. Calculation of Electrostatic Potential Energy

Electrostatic potential energy reflects the electrostatic interaction between a molecule and its neighbors [29]. The electrostatic interactions of CGA and its main amino acid active sites, aspartic acid (ASP), glutamine (GLN), and glutamate (GLU), were refined by an electrostatic field. GBVI/WSAdG scores were calculated and the potential energy with the highest score was selected as the final mode. The Gaussian 16 program was used for molecular optimization and theoretical calculations, and GaussView 6.0 was used for structural visualization. The electrostatic potential energy of the molecular surface was calculated using CubeGen and GaussView 6.0.

### 2.7. Analysis of the Bactericidal Activity of CGA on S. aureus in Pasteurized Milk

The bactericidal effect of CGA on *S. aureus* was determined using a method described by Sun et al. [30] with minor modifications. For this, *S. aureus* cells were diluted with pasteurized milk (Yunnan Eurasia Dairy Co., Ltd., Kunming, China) and counted to approximately 10^4^ CFU/mL. Thereafter, 2.5 mg/mL of CGA was added to the pasteurized milk and incubated at 4 °C for 7 d (*n* = 4). The number of colonies in pasteurized milk was determined by plate counting method to evaluate the effect of CGA on the growth of *S. aureus* in pasteurized milk.

### 2.8. Statistical Analysis 

All the experiments, except metabolomics, were performed in three independent replicates. Statistical analysis was performed in the IBM SPSS Statistic V20.0 software. Data are expressed as mean ± standard deviation (SD), and a *p*-value < 0.05 was considered as statistically significant. GraphPad Prism 5 was used for graphical evaluation.

## 3. Results

### 3.1. Antibacterial Effect of CGA on S. aureus in the Early Stage of Biofilm Formation

#### 3.1.1. The Effect of CGA on the MIC and Growth of *S. aureus*

**Figure 1:**
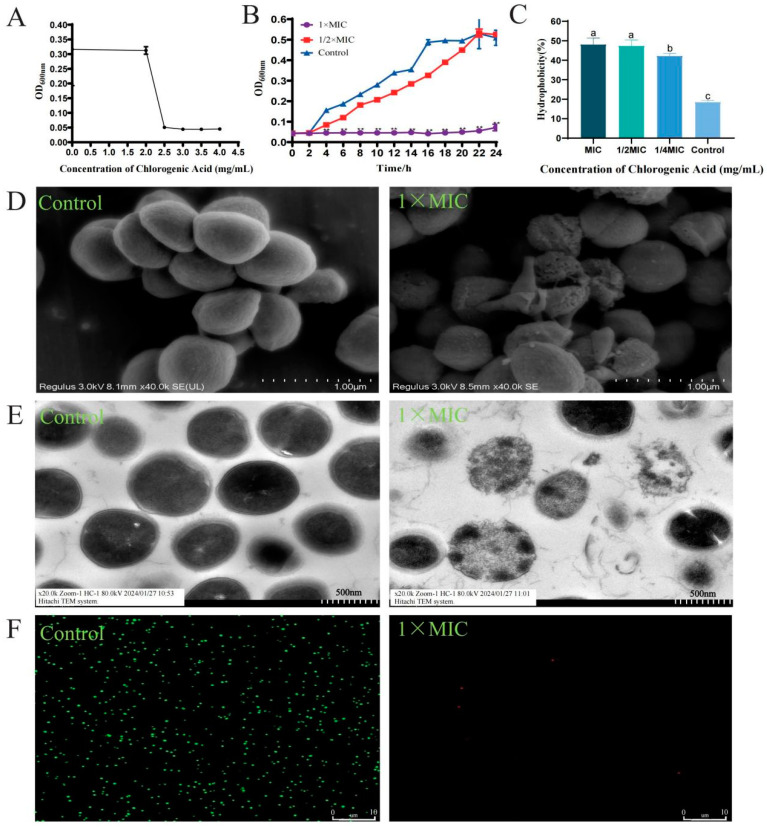
Antibacterial effect of CGA against *S. aureus* in the early stage of biofilm formation. (**A**) The MIC of CGA against *S. aureus* (** indicates a significant difference, *p* < 0.05). (**B**) Effect of CGA on the growth of *S. aureus*. (**C**) Effect of CGA on the surface hydrophobicity of *S. aureus* cells (different lowercase letters on the column indicate that the difference is statistically significant, *p* < 0.05). (**D**) SEM observation on the effect of CGA on the early biofilm of *S. aureus*. (**E**) TEM observation on the effect of CGA on the early biofilm of *S. aureus.* (**F**) Effect of CGA on the structure of early biofilm of *S. aureus*.

The results showed that CGA had antibacterial activity against *S. aureus*, and its MIC against *S. aureus* was 2.5 mg/mL (Figure 1A). To further explore the inhibitory effect of CGA on *S. aureus* in the initial stages of biofilm formation, we assessed the growth curve of *S. aureus* treated with 1.25 mg/mL and 2.5 mg/mL of CGA (Figure 1B). As shown in Figure 1B, CGA at 2.5 mg/mL completely inhibited *S. aureus* growth within 24 h, while CGA at 1.25 mg/mL reduced its viability.

#### 3.1.2. Effect of CGA on the Surface Hydrophobicity of *S. aureus* Cells

The hydrophobicity of the *S. aureus* groups treated with 0, 0.625 mg/mL, 1.25 mg/mL, and 2.5 mg/mL of CGA were 18.53%, 42.26%, 47.38%, and 48.16%, respectively, demonstrating that CGA induces a significant dose-dependent increase in the cell adsorption rates of *S. aureus* (*p* ≤ 0.05; Figure 1C).

#### 3.1.3. Effect of CGA on the Micromorphology of Early Biofilm of *S. aureus*

In this study, CGA was found to exhibit a dispersing effect on *S. aureus* biofilm. SEM and TEM were used to observe the effect of CGA on the microstructure of *S. aureus*. SEM observations showed that the untreated *S. aureus* biofilm had a smooth and round surface, intact bacterial cell morphology, strong intracellular adhesion, and dense structure (Figure 1D). In contrast, the CGA-treated *S. aureus* biofilms showed various morphological changes, bacterial cells were ruptured, intercellular adhesion was weakened, and the structure became loose (Figure 1D). TEM was conducted to observe the internal structure of *S. aureus* biofilms (Figure 1E). The results showed that the *S. aureus* cell wall membrane in the CGA-treated biofilm was blurred, showing serious damage and leakage. In addition, compared with the control group, the bacterial cells in the biofilm of the CGA treatment group were sparsely distributed and highly scattered.

#### 3.1.4. Effect of CGA on the Early Biofilm Structure of *S. aureus*

SYTO 9 and PI fluorescent probes were used to observe the effect of CGA on the early biofilms of *S. aureus*. SYTO 9 can freely penetrate the CM of all live cells to bind to nucleic acids and emits green fluorescence. In contrast, PI can only enter cells with damaged CM to bind to nucleic acids and emits red fluorescence [31]. As seen in Figure 1F, the cell slides without CGA treatment are full of green fluorescence and evenly distributed. There was almost no green fluorescence and a very small amount of red fluorescence on the cell slides treated with CGA, and the bacterial distribution was scattered. These results indicate that compared with the control group, CGA can increase the CM permeability of *S. aureus* and damage the cells. At the same time, the bacterial distribution is scattered, and the three-dimensional biofilm structure cannot be formed.

### 3.2. Inhibitory Effect of CGA on Cell Spreading and Biofilm Formation of S. aureus

#### 3.2.1. Effect of CGA on the Spreading of *S. aureus* Cells in the Early Stage of Biofilm Formation

**Figure 2:**
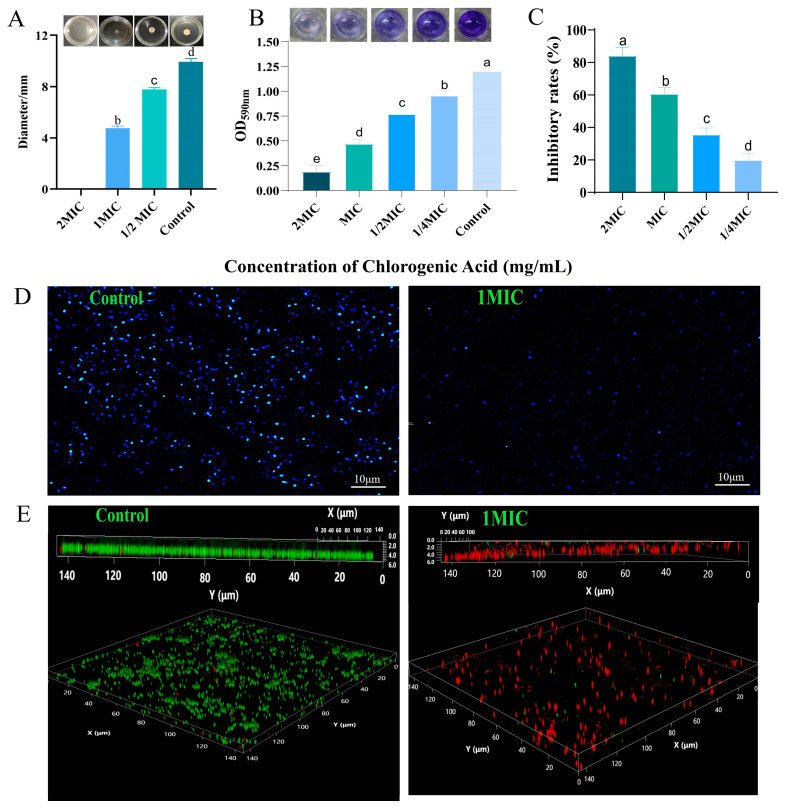
Inhibitory activity of CGA on *S. aureus* biofilm. (**A**) Effect of CGA on the spreading of *S. aureus* cells in the early stage of biofilm formation. (**B**) The inhibitory activity of different concentrations of CGA on *S. aureus* biofilm formation. (**C**) The inhibition rate of different concentrations of CGA on *S. aureus* biofilm formation. (**D**) DAPI staining of *S. aureus* biofilms. (**E**) CLSM images of *S. aureus* biofilms. Different lowercase letters on the column indicate that the difference is statistically significant, *p* < 0.05.

Spreading assay indicated that CGA inhibited the sliding of *S. aureus* in a dose-dependent manner as observed by a decrease in the distance moved by the bacteria in the presence of varying concentrations of CGA (*p* < 0.01) (Figure 2A).

#### 3.2.2. Effect of CGA on *S. aureus* Biofilm Formation

Figure 2 shows the effect of CGA on the biofilm formation of *S. aureus*. The results of crystal violet staining revealed that compared with the control group, the CGA-treated groups showed significantly reduced biofilm biomass under 5 mg/mL, 2.5 mg/mL, 1.25 mg/mL, and 0.625 mg/mL treatments (*p* < 0.05) in a dose-dependent manner (Figure 2B). *S. aureus* treated with 5 mg/mL of CGA showed an 84.1% inhibition rate of biofilm formation (Figure 2C).

#### 3.2.3. Effect of CGA on the Three-Dimensional Structure of *S. aureus* Biofilm

To study the effect of CGA on the morphological structure of *S. aureus* biofilm, the biofilms of CGA-treated and untreated *S. aureus* were observed under a fluorescence microscope. As shown in Figure 2D, the control group emitted a large amount of blue fluorescence, indicating that the biofilm of the untreated *S. aureus* contained a large number of bacteria, and the biofilm structure was complete and three-dimensional. In contrast, the CGA-treated groups showed significantly lower fluorescence intensity, decreased number of bacterial cells, and scattered (planktonic) cells on the slide surface.

The effect of CGA on the three-dimensional structure of *S. aureus* biofilm was observed by a CLSM. As shown in Figure 2E, the living bacteria on the cell slides without CGA treatment emitted a large area of bright green fluorescence. From the cross section of the biofilm, it grew closely, thick, had high activity, and had a good three-dimensional structure. In contrast, the CGA-treated *S. aureus* biofilm showed reduced green fluorescence and increased red fluorescence. Additionally, the CGA-treated *S. aureus* biofilm had a sparse and sporadic structure, was decomposed, had scattered cell distribution, and reduced three-dimensional rigidity.

### 3.3. Metabolomics-Enabled Discovery of Anti-Biofilm Pathways Mediated by CGA

**Figure 3:**
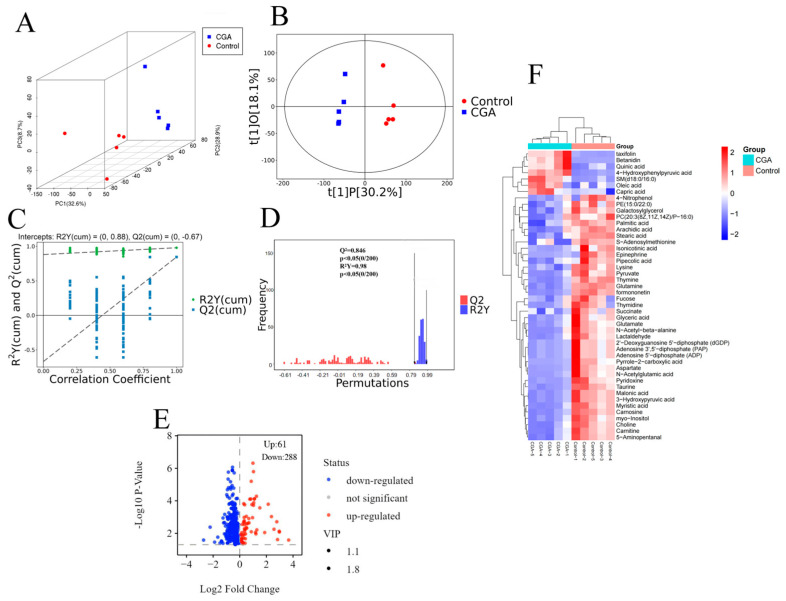
(**A**) Three-dimensional score scatter plot of PCA model. (**B**) The OPLS-DA model score scatter diagram. (**C**) The point diagram of the permutation test results of the OPLS-DA model. (The abscissa represents the permutation retention of the permutation test and the ordinate represents the R^2^Y or Q^2^ value. The green dot represents the R^2^Y value obtained by the permutation test, the blue square point represents the Q^2^ value obtained by the permutation test, and the two dashed lines represent the regression lines of R^2^Y and Q^2^.) (**D**) The histogram of permutation test results of the OPLS-DA model. (**E**) Volcano plot of the 349 differential metabolites between the control and CGA-treated groups. (**F**) Heat map of the 46 differential metabolites between the control and CGA-treated groups.

Non-targeted metabolomics showed that there was a significant separation between CGA treatment and control samples, indicating that there was a significant metabolic response to CGA treatment (Figure 3A). These results were further confirmed by orthogonal partial least squares discriminant analysis (OPLS-DA), which demonstrated a notable distinction in the metabolite profiles of the two groups (Figure 3B). A total of 4123 metabolites were identified. The permutation test of the OPLS-DA model confirmed the significance of the metabolic changes, with R^2^Y and Q^2^ values indicating the model’s predictability and reliability (Figure 3C,D).

The differential metabolites were screened using Student’s *t*-test (*p* < 0.05 and VIP > 1) in the OPLS-DA model. Compared to the control group, a total of 349 (288 down-regulated [blue] and 61 up-regulated [red]) differential metabolites were identified in the CGA-treated groups, which are presented in the volcano plot in Figure 3E. These differential metabolites were imported into the KEGG database for identification, and 46 differential metabolites matching the KEGG ID were subjected to hierarchical clustering analysis (HCA). Among these, 39 and 7 differential metabolites were down-regulated and up-regulated, respectively, in the CGA-treated group compared with in the control group (Figure 3F). Results of HCA showed a significant difference between the control and CGA-treated groups, consistent with the results of PCA and OPLS-DA.

**Figure 4:**
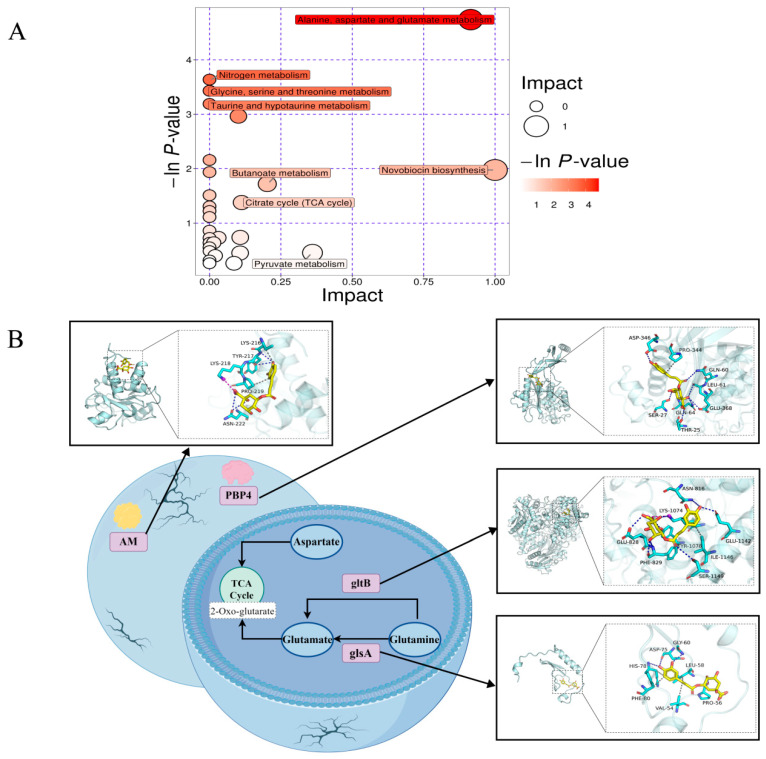
(**A**) Metabolic pathway enrichment analysis of the differential metabolites in the control and CGA-treated groups. (**B**) Molecular docking analysis of CGA and key enzymes involved in biofilm formation.

Metabolic pathway enrichment analysis of the 46 differential metabolites was conducted using KEGG and MetaboAnalyst (Table 1). The results showed that the differential metabolites were primarily enriched in amino acid metabolism (alanine, aspartate, and glutamic acid metabolism; glycine, serine, and threonine metabolism, etc.); membrane transport (ABC transporter); nitrogen metabolism; and taurine and hypotaurine metabolism. The most significant metabolic pathways were screened according to the *p*-value (< 0.05) and presented as bubble plots (Figure 4A). As seen in Figure 4A, CGA treatment significantly disrupted the amino acid metabolic pathway of *S. aureus.*

### 3.4. The Role of Amino Acid Metabolic Pathway in Biofilm Formation of S. aureus

#### 3.4.1. Analysis of Amino Acid Metabolic Pathway of *S. aureus*

The results of metabolomics showed that the metabolism of ASP, GLN, and GLU in the CGA treatment group was significantly down-regulated, mainly involving amino acid metabolic pathways such as alanine, aspartic acid, glutamic acid metabolism, glycine, serine, and threonine metabolism. It is speculated that CGA treatment leads to the disorder of these amino acids in the biofilm of *S. aureus.*

#### 3.4.2. Targeting of Multiple Proteins for Biofilm Inhibition

The specific binding modes of CGA with gltB, glsA, AM, and PBP4 were determined to elucidate the molecular basis of its influence on *S. aureus* biofilm stability (Figure 4B). The results showed that CGA can form H-bonds with TYR-217 and ASN-222, a salt bridge with LYS-218, and hydrophobic interaction with LYS-216 and PRO-219 of AM protein. Additionally, CGA can form H-bonds with ASP-346, GLN-64, SER-27, GLU-368, LEU-61, THR-25, and GLN-60 and hydrophobic interaction with PRO-344 of PBP4. In addition, CGA formed H-bonds with PHE-829, LYS-1074, SER-1149, GLU-828, GLU-1142, ASN-816, and TYR-1078 and a salt bridge with LYS-1074 of gltB protein. Moreover, it forms hydrophobic interactions with LYS-1074 and ILE-1146 and H-bonds with HIS-78, PRO-56, ASP-75, and GLY-60 of glsA protein. The hydrophobic interactions provide a strong van der Waals force for the molecule, while the H-bonds and salt bridges improve the binding between the protein and CGA. The negative binding affinity indicates the possibility of binding. In this study, the binding affinities of CGA with AM, PBP4, gltB, and glsA proteins were found to be −6.525, −6.797, −6.915, and −7.086 kcal/mol, respectively.

#### 3.4.3. Potential Analysis of the Combination of CGA and Amino Acids

**Figure 5:**
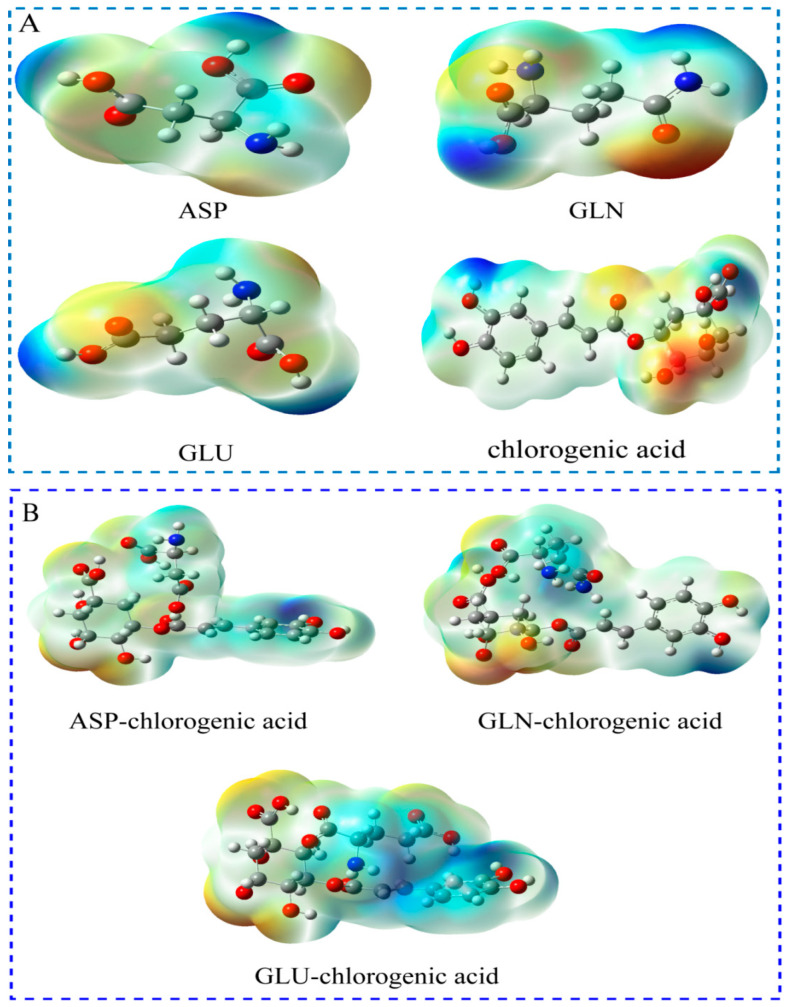
(**A**) Single-molecule electrostatic potential energy distribution maps of CGA and ASP, GLN, and GLU. (**B**) The electrostatic potential energy distribution of CGA and ASP, GLN, and GLU. Blue represents the positive potential region, and red represents the negative potential region.

Docking studies revealed that CGA binds effectively to ASP, GLN, and GLU with binding energies of −3.14, −3.27, and −3.29 kcal/mol, respectively (Figure 5). The binding sites are located primarily near the hydroxyl (-OH) and carbonyl (-C=O) groups, indicating nucleophilic and electrophilic interactions.

### 3.5. Extending the Shelf Life of Pasteurized Milk with CGA

**Figure 6:**
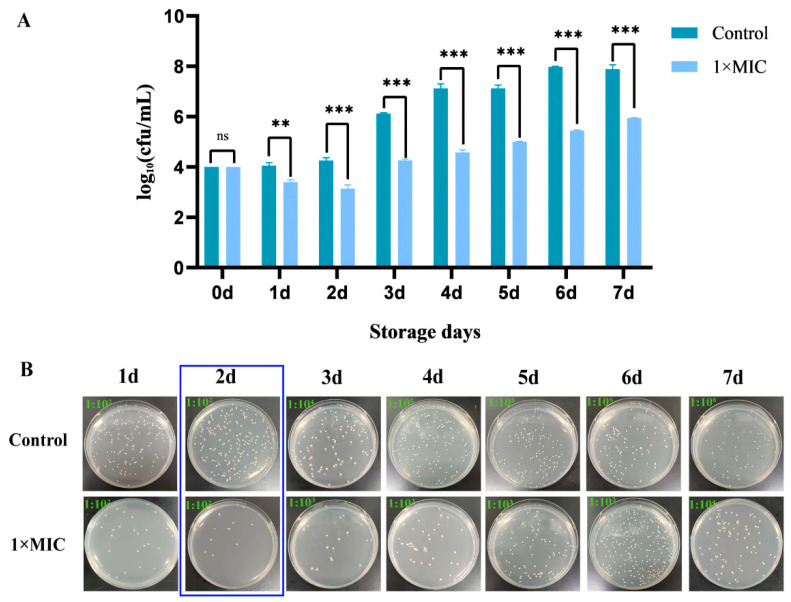
Effect of CGA on *S. aureus* in pasteurized milk. (**A**) The inhibitory effect of CGA on *S. aureus* in pasteurized milk. (**B**) The appearance map of CGA treatment (the upper right corner indicates the dilution ratio, ns, *p* > 0.05, ** *p* ≤ 0.01 and *** *p* ≤ 0.001, compared with the control group).

In this study, we prepared *S. aureus*-contaminated pasteurized milk to verify the inhibitory effects of CGA on *S. aureus* activity and biofilm-forming ability in dairy products. The results showed that the total number of colonies in the untreated milk (control) exceeded the safe detection limit of 6.00 log_10_ CFU/mL after 3 d of incubation at 4 °C and continued to increase in the following days (Figure 6A). In contrast, treatment with 2.5 mg/mL of CGA significantly decreased the number of bacteria in the milk within 48 h. Although *S. aureus* continued to grow in the milk, the log_10_ CFU/mL value was significantly lower in the CGA-treated milk compared to in the control milk (*p* ≤ 0.001), being < 6.00 log_10_ CFU/mL, thus meeting the safety test standard. In addition, assessments were conducted and visual effects visualized. The pasteurized milk sample is shown in Figure 6B. After pasteurized milk contaminated by *S. aureus* was treated with 2.5 mg/mL CGA, the number of colonies was significantly less than that of the control group.

## 4. Discussion

In recent years, biofilm formation is still a worldwide public health concern, especially in relation to foodborne diseases. With the excessive use of antibiotics for a long time, multidrug-resistant bacteria continue to appear, the effectiveness of traditional antibiotics is reduced, new antibiotics are slow to develop, bacteria are prone to drug resistance, and there are certain safety hazards. Therefore, it is urgent to find natural green antibacterial substances. It was found that eugenol could be used as a potential anti-biofilm agent to inhibit the biofilm formation at the air–liquid interface of *Bacillus amyloliquefaciens* [32]. In addition, (+) -nocardone (a sesquiterpene ketone found in common grapefruit) was reported to have a good anti-biofilm effect on multidrug-resistant *S. aureus* [33]. Several studies reported that CGA has excellent antibacterial activity. For instance, Lou et al. reported that the MIC of CGA against *Bacillus subtilis* was 40 μg/mL [13]. Su et al. reported that it has an MIC of 5 mg/mL against *Pseudomonas fluorescens* and *Staphylococcus saprophyticus*, isolated from chicken [34]. Li et al. [12] showed that CGA has an MIC of 2.5–5.0 mg/mL against *S. aureus*, consistent with the results of our study. These results indicate that CGA has an antibacterial effect against *S. aureus*, which can affect its initial biofilm adhesion and inhibit its biofilm formation. It is a natural antibacterial agent worthy of development.

A previous study found that after 48 h of formation, biofilm reaches the mature stage, wherein its three-dimensional structure is fully formed and the bacterial cells on the surface remain active [35]. These results indicate that CGA has an inhibitory effect on the growth of *S. aureus* within the first 24 h of biofilm maturation, thereby inhibiting the formation and maturation of biofilms in the subsequent 24 h. Liŭ et al. [36] found that the bactericidal effect of dihydromyricetin against *Vibrio parahaemolyticus* was positively correlated with the increase in bacterial surface hydrophobicity, which further affects its bacteriostatic effect. Cell surface hydrophobicity is closely related to bacterial adhesion and biofilm formation [36]. Our results showed that CGA significantly affected the hydrophobicity of the bacterial surface to achieve an antibacterial effect, while reducing bacterial adhesion and inhibiting biofilm synthesis.

Through SEM observations, Li et al. [12] found that *S. aureus* cells treated with CGA had broken CM and a deformed CW. A previous study demonstrated that CGA can disrupt the CM integrity and structure of *Yersinia enterocolitica*, thereby increasing CM permeability and leakage of intracellular components, ultimately leading to its death [9]. CGA destroyed the cell membrane of *S. aureus* and led to the outflow of contents, and the cell distribution became loose and scattered. Studies have shown that CGA can reduce the content of OM lipopolysaccharide, disrupt the ratio of OM lipopolysaccharide–phospholipid, induce OM shedding, and eventually lead to *Salmonella typhimurium* death [37]. These results suggest that CGA may inhibit the formation and maintenance of *S. aureus* biofilm by interfering with the interaction between bacterial cells and the extracellular matrix, making it difficult for residual bacterial cells to re-adhere and leading to biofilm scattering.

CGA treatment caused damage to *S. aureus*. Similar to the report of MEL-A treatment of *S. aureus* biofilm, MEL-A treatment reduced the rigidity of the three-dimensional structure of biofilm [2]. Fluorescence microscopy and CLSM observation showed that CGA could penetrate biofilm and destroy biofilm components, thereby killing bacterial cells, reducing biofilm thickness, and destroying its three-dimensional structure. At the same time, amino acid metabolism was very active during the formation process of *S. aureus* biofilm. In this study, we found that CGA significantly affected the metabolism of ASP, GLN, and GLU in *S. aureus*. Studies have shown that bacteria respond to environmental stimuli by regulating amino acid metabolic pathways. The results of metabolomics showed that the metabolism of ASP, GLN, and GLU in CGA treatment group was significantly down-regulated, and the abnormal changes in GLU and ASP metabolism may also lead to the abnormality of the ABC transport system [38]. It is speculated that the destruction of the ABC transport system hinders the transport of intracellular substances, reducing cell adhesion. The destruction of the ABC transport system hinders the transport of intracellular substances, reduces cell adhesion, disperses the biofilm, and inhibits the formation of the biofilm.

Molecular docking and electrostatic potential analysis showed that CGA can bind to metabolism-related proteins (glt B, gls A), cell wall membrane synthesis-related proteins (AM protein, PBP4), and metabolism-related amino acids (ASP, GLN, GLU). *S. aureus* biofilm formation is mediated by the CW hydrolase-AM protein, which is overexpressed during biofilm formation [39]. CGA can interact with AM, leading to the degradation and remodeling of the CW, which is critical for biofilm formation and diffusion. PBP4 is a transpeptidase involved in the cross-linking of peptidoglycan and increases the structural rigidity of the CW [40]. Therefore, it is speculated that CGA affects the synthesis of glutamic acid by interfering with glt B and gls A, resulting in the down-regulation of amino acids. Interfering with CGA-mediated AM and PBP4 functions may change the degree of cross-linking and hardness of the CW, thereby affecting the stability of the biofilm. The binding sites are mainly located near the hydroxyl (-OH) and carbonyl (-C=O) groups, indicating nucleophilic and electrophilic interactions. These functional groups are close to each other between the molecules, which is conducive to the formation of strong hydrogen bonds and electrostatic interactions. The effective combination of CGA with ASP, GLN, and GLU further explains the down-regulation of these three amino acids and their metabolic disorders. It is speculated that the strong electrostatic interaction between CGA and ASP, GLN, and GLU leads to the apparent membrane rupture, inhibition, and scattering of *S. aureus* biofilm.

*S. aureus* DC.RB-015 has shown resistance to a variety of antibiotics, including penicillin, ofloxacin, erythromycin, clindamycin, tetracycline, and cefoxitin. The strain also has enhanced biofilm formation, which may further enhance its resistance to antibiotics. Given these properties, the MIC of CGA against *S. aureus* DC.RB-015 is expected to be higher than the MIC against normal strains [3]. Some similar previous studies have also reported the MIC values of CGA against *S. aureus*, *Pseudomonas aeruginosa*, and multidrug-resistant *Klebsiella pneumoniae*. The MIC value of CGA against *S. aureus* was 2.5–5.0 mg/mL [12], the MIC value of *Pseudomonas aeruginosa* was 5 mg/mL [41], and the MIC value of multidrug-resistant *Klebsiella pneumoniae* was 4 mg/mL [42]. The MIC of CGA against *S. aureus* in this study is in the middle of this range. In addition, CGA has a slightly bitter, less sour taste and does not affect the sensory quality of milk at the MIC value [43]. As a new dosage form, CGA has been tested and used in many clinical trials with high safety and poses no harm to humans at low doses [44]. Children are at a crucial stage of growth and development, with different physiological and metabolic profiles to adults. It is therefore important to monitor their daily milk intake. Consumption of more than 1 L of milk per day could result in an intake of approximately 2.5 g of CGA in children.

In conclusion, CGA has a certain inhibitory effect on the initial formation of *S. aureus* biofilm. It inhibits bacterial adhesion by destroying the integrity of the bacterial CM, resulting in increased hydrophobicity and significant changes in morphology. CGA specifically targets key players in amino acid metabolism, such as PBP4, AM, gltB, and glsA, as well as essential amino acids, such as ASP, GLN, and GLU, thereby affecting the structural integrity and adhesion of biofilms. The dual mechanism of CGA targeting both membrane integrity and biofilm-related proteins and amino acids makes it a promising and environmentally friendly biofilm control preservative.

## Figures and Tables

**Table 1:** Metabolic pathways of the 46 differential metabolites in the *S. aureus* cells treated with 2.5 mg/mL of CGA.

Serial Number	Metabolite	KEGG Metabolic Pathway	Up-Regulation or Down-Regulation
1	Glutamine	Arginine biosynthesis Alanine, aspartate, and glutamate metabolism D-Amino acid metabolism Glyoxylate and dicarboxylate metabolism Nitrogen metabolism Biosynthesis of amino acids ABC transporters Two-component system	Down-regulation
2	Glutamate	Arginine biosynthesis Alanine, aspartate, and glutamate metabolism Arginine and proline metabolism Histidine metabolism Taurine and hypotaurine metabolism D-Amino acid metabolism E-Nitrogen metabolism Biosynthesis of alkaloids derived from ornithine, lysine, and nicotinic acid Metabolic pathways Biosynthesis of amino acids ABC transporters Two-component system	Down-regulation
3	Aspartate	Arginine biosynthesis Alanine, aspartate, and glutamate metabolism Glycine, serine, and threonine metabolism Cysteine and methionine metabolism Lysine biosynthesis Histidine metabolism Beta-alanine metabolism Cyanoamino acid metabolism D-Amino acid metabolism E-Biosynthesis of alkaloids derived from ornithine, lysine, and nicotinic acid Metabolic pathways Biosynthesis of amino acids ABC transporters Two-component system	Down-regulation
4	Thymine	Pyrimidine metabolism Metabolic pathways Nucleotide metabolism	Down-regulation
5	Carnosine	Histidine metabolism Beta-alanine metabolism Metabolic pathways	Down-regulation
6	Malonic acid	Fatty acid biosynthesis Pyrimidine metabolism Beta-alanine metabolism Metabolic pathways Fatty acid metabolism	Down-regulation
7	3-Hydroxypyruvic acid	Glycine, serine, and threonine metabolism Metabolic pathways	Down-regulation
8	Myristic acid	Fatty acid biosynthesis Metabolic pathways	Down-regulation
9	Lysine	Lysine biosynthesis Lysine degradation D-Amino acid metabolism Biosynthesis of alkaloids derived from ornithine, lysine, and nicotinic acid 2-Oxocarboxylic acid metabolism Biosynthesis of amino acids ABC transporters Protein digestion and absorption	Down-regulation
10	Stearic acid	Fatty acid biosynthesis Metabolic pathways	Down-regulation
11	5-Aminopentanal	Lysine degradation Metabolic pathways	Down-regulation
12	Pyruvate	Glycolysis/Gluconeogenesis Citrate cycle (TCA cycle) Pentose phosphate pathway Pentose and glucuronate interconversions Alanine, aspartate, and glutamate metabolism Glycine, serine, and threonine metabolism Cysteine and methionine metabolism Valine, leucine, and isoleucine biosynthesis Arginine and proline metabolism Tyrosine metabolism Phenylalanine metabolism Benzoate degradation Taurine and hypotaurine metabolism D-Amino acid metabolism	Down-regulation
13	Taurine	Taurine and hypotaurine metabolism Metabolic pathways ABC transporters	Down-regulation
14	Carnitine	Thermogenesis bile secretion Diabetic cardiomyopathy	Down-regulation
15	SM(d18:0/16:0)	Sphingolipid metabolism Metabolic pathways Sphingolipid signaling pathway	Up-regulation
16	Choline	Glycine, serine, and threonine metabolism Teichoic acid biosynthesis Glycerophospholipid metabolism Metabolic pathways ABC transporters	Down-regulation
17	Myo-Inositol	Metabolic pathways Biosynthesis of nucleotide sugars ABC transporters	Down-regulation
18	Arachidic acid	Biosynthesis of unsaturated fatty acids	Down-regulation
19	Galactosylglycerol	Galactose metabolism Glycerolipid metabolism	Down-regulation
20	Lactaldehyde	Fructose and mannose metabolism Pyruvate metabolism Propanoate metabolism Metabolic pathways	Down-regulation
21	Quinic acid	Phenylalanine, tyrosine, and tryptophan biosynthesis Metabolic pathways	Up-regulation
22	N-Acetyl-beta-alanine	Beta-alanine metabolism	Down-regulation
23	Taxifolin	Metabolic pathways	Up-regulation
24	Pyridoxine	Metabolic pathways	Down-regulation
25	PE(15:0/22:0)	Glycerophospholipid metabolism Metabolic pathways	Down-regulation
26	Betanidin	Betalain biosynthesis Betalain WAF Metabolic pathways	Up-regulation
27	Formononetin	Isoflavonoid biosynthesis Biosynthesis of phenylpropanoids Metabolic pathways Biosynthesis of secondary metabolites	Down-regulation
28	Palmitic acid	Fatty acid biosynthesis Fatty acid elongation Fatty acid degradation Metabolic pathways Fatty acid metabolism	Down-regulation
29	Glyceric acid	Pentose phosphate pathway Glycine, serine, and threonine metabolism Glycerolipid metabolism Metabolic pathways	Down-regulation
30	Pipecolic acid	Lysine degradation Biosynthesis of alkaloids derived from ornithine, lysine, and nicotinic acid Metabolic pathways	Down-regulation
31	4-Hydroxyphenylpyruvic acid	Tyrosine metabolism Phenylalanine, tyrosine, and tryptophan biosynthesis Metabolic pathways 2-Oxocarboxylic acid metabolism Biosynthesis of amino acids	Up-regulation
32	N-Acetylglutamic acid	Arginine biosynthesis D-Amino acid metabolism Metabolic pathways Biosynthesis of amino acids	Down-regulation
33	S-Adenosylmethionine	Cysteine and methionine metabolism Arginine and proline metabolism Metabolic pathways Biosynthesis of amino acids	Down-regulation
34	Adenosine 5′-diphosphate (ADP)	Purine metabolism Metabolic pathways Nucleotide metabolism	Down-regulation
35	2′-Deoxyguanosine 5′-diphosphate (dGDP)	Purine metabolism Metabolic pathways Nucleotide metabolism	Down-regulation
36	Adenosine 3′,5′-diphosphate (PAP)	Purine metabolism Sulfur metabolism Metabolic pathways	Down-regulation
37	Capric acid	Fatty acid biosynthesis Metabolic pathways	Up-regulation
38	Pyrrole-2-carboxylic acid	D-Amino acid metabolism	Down-regulation
39	PC(20:3(8Z,11Z,14Z)/P-16:0)	Glycerophospholipid metabolism Arachidonic acid metabolism Linoleic acid metabolism Alpha-linolenic acid metabolism Metabolic pathways	Down-regulation
40	Succinate	Citrate cycle (TCA cycle) Oxidative phosphorylation Alanine, aspartate, and glutamate metabolism Lysine degradation Tyrosine metabolism Phenylalanine metabolism Pyruvate metabolism Biosynthesis of alkaloids derived from ornithine, lysine, and nicotinic acid Metabolic pathways Two-component system	Down-regulation
41	Isonicotinic acid	Drug metabolism—other enzymes	Down-regulation
42	Epinephrine	Tyrosine metabolism Metabolic pathways Two-component system Quorum sensing	Down-regulation
43	Thymidine	Pyrimidine metabolism Metabolic pathways Nucleotide metabolism	Down-regulation
44	4-Nitrophenol	Microbial metabolism in diverse environments	Down-regulation
45	Oleic acid	Fatty acid biosynthesis	Up-regulation
46	Fucose	Fructose and mannose metabolism Amino sugar and nucleotide sugar metabolism Metabolic pathways Biosynthesis of nucleotide sugars Two-component system Quorum sensing	Down-regulation

## Data Availability

The original contributions presented in the study are included in the article, further inquiries can be directed to the corresponding authors.
